# Applying an explainable machine learning model might reduce the number of negative appendectomies in pediatric patients with a high probability of acute appendicitis

**DOI:** 10.1038/s41598-024-63513-x

**Published:** 2024-06-04

**Authors:** Ivan Males, Zvonimir Boban, Marko Kumric, Josip Vrdoljak, Karlotta Berkovic, Zenon Pogorelic, Josko Bozic

**Affiliations:** 1 Department of Abdominal Surgery, University Hospital of Split, Spinciceva 1, 21000 Split, Croatia; 2 Department of Medical Physics and Biophysics, School of Medicine, University of Split, Soltanska 2A, 21000 Split, Croatia; 3 Department of Pathophysiology, School of Medicine, University of Split, Soltanska 2A, 21000 Split, Croatia; 4 Laboratory for Cardiometabolic Research, School of Medicine, University of Split, Soltanska 2A, 21000 Split, Croatia; 5 Department of Surgery, School of Medicine, University of Split, Soltanska 2A, 21000 Split, Croatia; 6 Department of Pediatric Surgery, University Hospital of Split, Spinciceva 1, 21000 Split, Croatia

**Keywords:** Acute appendicitis, Appendectomy, Machine learning, Pediatric surgery, Children, Gastroenterology, Medical research

## Abstract

The diagnosis of acute appendicitis and concurrent surgery referral is primarily based on clinical presentation, laboratory and radiological imaging. However, utilizing such an approach results in as much as 10–15% of negative appendectomies. Hence, in the present study, we aimed to develop a machine learning (ML) model designed to reduce the number of negative appendectomies in pediatric patients with a high clinical probability of acute appendicitis. The model was developed and validated on a registry of 551 pediatric patients with suspected acute appendicitis that underwent surgical treatment. Clinical, anthropometric, and laboratory features were included for model training and analysis. Three machine learning algorithms were tested (random forest, eXtreme Gradient Boosting, logistic regression) and model explainability was obtained. Random forest model provided the best predictions achieving mean specificity and sensitivity of 0.17 ± 0.01 and 0.997 ± 0.001 for detection of acute appendicitis, respectively. Furthermore, the model outperformed the appendicitis inflammatory response (AIR) score across most sensitivity–specificity combinations. Finally, the random forest model again provided the best predictions for discrimination between complicated appendicitis, and either uncomplicated acute appendicitis or no appendicitis at all, with a joint mean sensitivity of 0.994 ± 0.002 and specificity of 0.129 ± 0.009. In conclusion, the developed ML model might save as much as 17% of patients with a high clinical probability of acute appendicitis from unnecessary surgery, while missing the needed surgery in only 0.3% of cases. Additionally, it showed better diagnostic accuracy than the AIR score, as well as good accuracy in predicting complicated acute appendicitis over uncomplicated and negative cases bundled together. This may be useful in centers that advocate for the conservative treatment of uncomplicated appendicitis. Nevertheless, external validation is needed to support these findings.

## Introduction

Acute appendicitis represents one of the most common surgical emergencies, especially in pediatric patients presenting with abdominal pain^1,2^. The incidence is relatively stable at 1 per 1000 per year in Western countries, whereas a trend towards an increase in incidence is observed in newly industrialized countries^3^. Although making the diagnosis is usually straightforward in patients presenting with classical symptoms, such as migrating abdominal pain and nausea, atypical presentations commonly lead to unnecessary explorations and delays in treatment^4,5^. Burdened by the historical data suggesting high mortality and morbidity in untreated appendicitis, management of suspected appendicitis has been based for a long time on the principle of early exploration of wide indications to prevent perforation^6^.

A prevailing opinion is that untreated appendicitis will eventually progress to perforation thus causing inevitable worsening in clinical outcomes^7^. Accordingly, medical professionals use advanced diagnostic modalities, such as ultrasound, computerized tomography (CT) and magnetic resonance imaging (MRI) to correctly identify this pathology.

However, available data suggests that spontaneous resolution of appendicitis is more common than previously thought and that delay in treatment after hospital admission doesn’t increase rates of perforation^8^. Moreover, insights in the large registries of appendectomies point out that although perforation is associated with increased mortality, mortality rates of negative appendectomies in patients undergoing surgery for non-specific abdominal pain are also increased beyond what could be explained by an underlying condition concealed by the appendectomy^9^.

In the absence of consistent international guidelines, the diagnosis of appendicitis and concurrent surgery referral is mostly based on clinical data, standard laboratory findings, and ultrasound^10^. The diagnostic accuracy of such an approach can vary significantly depending on sex, race, and hospital experience, but overall, the incidence of negative appendectomies is around 10–15%^11^. Several risk scores were developed to aid in the diagnosis of acute appendicitis in a pediatric population: the appendicitis inflammatory response score (AIR), Alvarado score, pediatric appendicitis score (PAS), and the pediatric appendicitis risk calculator (pARC)^12^. AIR and pARC seem to offer higher specificity and positive predictive values compared to the Alvarado and the PAS scores, yet, the rate of negative appendectomies remains relatively high even with the use of the aforementioned scores^13^.

Machine learning (ML) recently emerged as a useful modality for the improvement of management strategies in virtually all fields of medicine^14^. The created learning models leverage large amounts of data to extract complex statistical patterns with predictive power superior to that of standard modalities^15^. The usefulness of ML models is largely dependent on the amount and quality of data on which it is trained, but also on the adequate setting of model accuracy.

The primary aim of the present study was to develop an ML model which can reduce the number of negative appendectomies in pediatric patients with a high clinical probability of acute appendicitis. A secondary aim was to construct a model that would differentiate patients with complicated acute appendicitis from patients who either have uncomplicated appendicitis or no appendicitis.

## Materials and methods

### Study design and ethical considerations

The data for this study were gathered from the patient’s records from the department of pediatric surgery, University Hospital of Split, with suspected acute appendicitis who underwent appendectomy. The data was gathered from January 2019 to July 2023. The Ethics Committee of the University Hospital of Split approved the study protocol (approval number 500-03/22-01/188; date of approval November 28, 2022) conforming to the World Health Organization Declaration of Helsinki of 1975 as revised in 2013, and the International Conference on Harmonization Guidelines on Good Clinical Practice. The patients’ anonymity was strictly maintained.

### Inclusion and exclusion criteria

All pediatric patients (0–17 years of age) that were referred to urgent surgical treatment under assumption of acute appendicitis were included in the study. Exclusion criteria were age > 17 years, the presence of any significant comorbidity (chronic cardiac, renal, or gastrointestinal condition), body mass index (BMI) ≥ 35 kg/m^2^, incidental appendectomy, and PHD that is neither appendicitis nor histologically normal appendix (e.g. neuroendocrine tumors or enterobiasis) or if no histopathology report was available.

### Study aims and data preparation

We defined three study goals:

- Development of a model for the prediction of negative and positive acute appendicitis cases based on the PHD.
- Comparison of performance of the appendicitis prediction model to the acute (AIR) score.
- The differentiation between complicated from uncomplicated appendicitis cases and negative appendectomies.

The initial set of features contained patient data, features from complete and differential blood counts, biochemistry measures (such as sodium concentration and C-reactive protein—CRP), and clinical examination features (presence of abdominal pain and rebound tenderness or guarding). All patients underwent surgical treatment, which in the majority of cases was done as three port laparoscopic appendectomy. In very few cases standard open appendectomy was performed. Operative technique was chosen on surgeon’s preference.

Twenty-two features were included for model training and analysis: age, sex, symptoms duration, height, weight, BMI, body temperature, white blood cells (WBC) count, CRP, neutrophil percentage, lymphocyte percentage, thrombocyte/lymphocyte ratio (TLR), neutrophil/lymphocyte ratio (NLR), mean platelet volume (MPV), mean corpuscular hemoglobin concentration (MCHC), sodium concentration, rebound tenderness, and the presence or absence of signs and symptoms such as vomiting, nausea, and pain migration. The outcome feature was PHD. Patients with confirmed pathohistological diagnosis (PHD) of acute appendicitis were subdivided as having either uncomplicated appendicitis (catarrhal or phlegmonous) or complicated appendicitis (gangrenous or gangrenous-perforated) based on histopathology reports.

Features were excluded if more than 30% of data was missing and/or they were highly correlated with some of the chosen features, such as hemoglobin count, hematocrit, mean corpuscular hemoglobin, etc.

In total, the dataset consisted of 614 pediatric patients. Patients were excluded if more than two missing values were present among the features that were determined to be highly important based on previous studies: neutrophils percentage, lymphocytes count, WBC count, CRP, and sodium concentration features. After applying the above exclusion criteria, 551 patients was included in the final analysis. Among 551 patients, 47 cases were negative for appendicitis, 252 had uncomplicated appendicitis, and 252 had complicated appendicitis, indicating an imbalanced dataset. The final dataset contained missing values as presented in Table 1.

**Table 1:** Features and percentage of missing values.

Feature	Percentage of missing values
TLR	29.2
Lymphocytes percentage	29
NLR	29
Sodium concentration	24.5
Height	11.4
Weight	11.4
BMI	11.4
RDW	8.5
MPV	8.5
MCHC	7.6
Thrombocytes	5.3
Neutrophils percentage	5.1
Body temperature	3.4
Nausea	3.4
Migration	3.1
Vomiting	1.5
Symptom duration	1.1
Rebound tenderness	0.9
Age	0
Sex	0
WBC count	0
CRP	0

*TLR* thrombocyte to lymphocyte ratio, *NLR* neutrophil to lymphocyte ratio, *BMI* body mass index, *RDW* red blood cell distribution width, *MPV* mean platelet volume, *MCHC* mean corpuscular hemoglobin concentration, *CRP* C-reactive protein, WBC white blood cell.

### Prediction model training, optimization, and validation

We tested three ML algorithms: random forest, eXtreme gradient boosting (XGBoost), and Logistic regression. The first two algorithms were chosen based on their known effectiveness in dealing with tabular data and imbalanced datasets, while logistic regression was chosen as a baseline model^16–18^.

**Figure 1:**
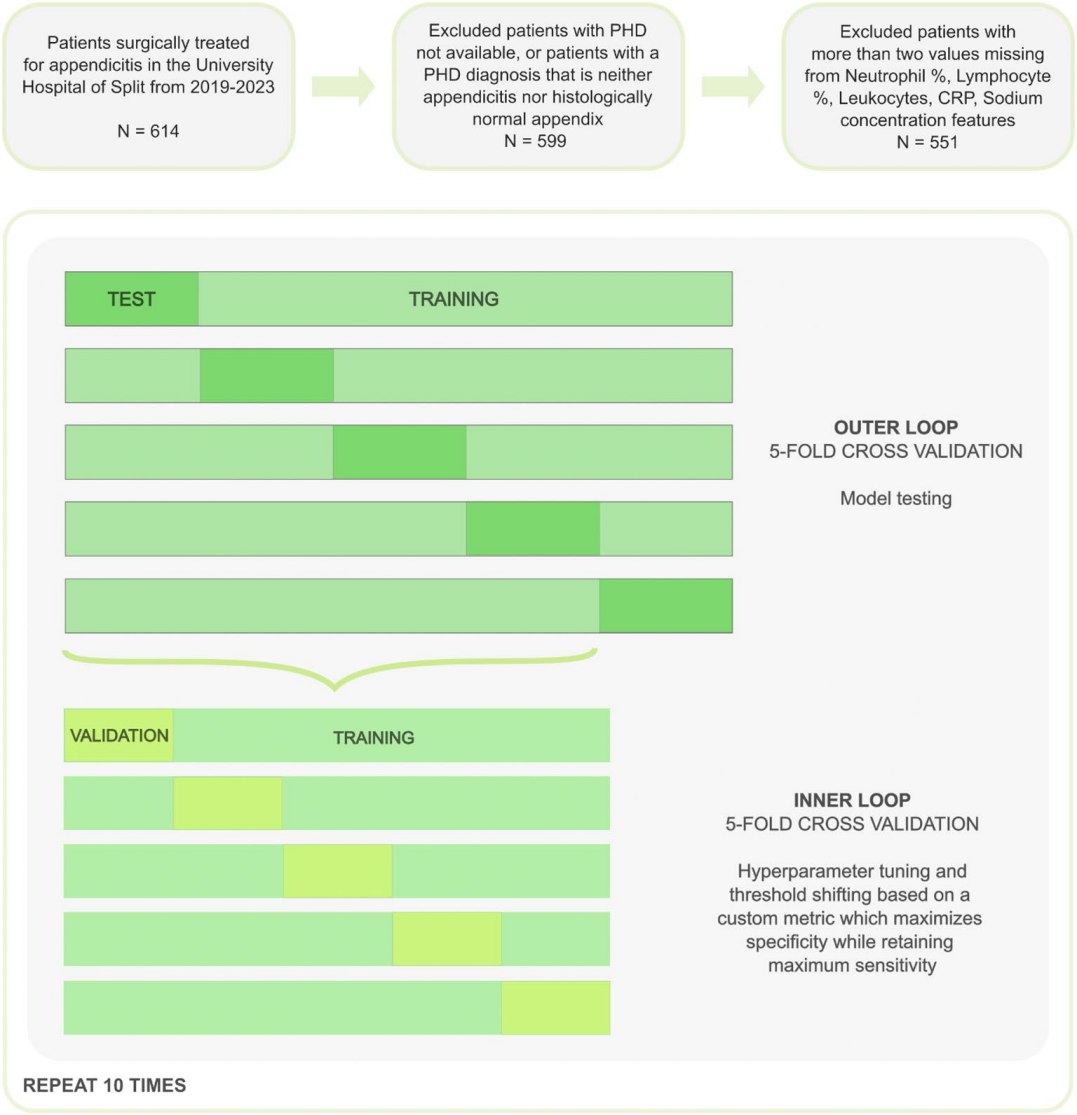
Study workflow (feature selection, model training, and validation).

For model training and validation, the nested cross-validation approach was used, with five-fold inner and outer cross-validation, repeated 10 times (Fig. 1). Consequently, each outer fold was split into training (80%) and test sets (20%), with stratification on the target variables due to the imbalanced nature of the dataset. Inner cross-validation was performed on each outer fold’s training set to tune the hyperparameters and perform threshold shifting. Missing values were imputed by utilizing the Bagged Trees algorithm (using the “step_impute_bag” function from the “recipes” package in the R programming language). To avoid data leakage, the imputation of the validation and test datasets was performed only based on estimates calculated from their training counterparts.

Threshold shifting on the receiver operator characteristic curve (ROC) was used to address the data imbalance. Since our goal was to build a model that improves the detection of negative cases without sacrificing the ability to detect positive cases, we chose to tune our model hyperparameters and perform threshold shifting based on a custom metric that locates the point of highest specificity on the ROC curve for which sensitivity is still 1 (max (specificity/sensitivity) = 1).

The threshold applied to the test set is determined as the second lowest threshold obtained from inner cross-validation. In terms of retaining high sensitivity on the test set, this is a more conservative approach compared to taking the mean threshold. In other words, we imposed a constraint that the model should be 100% accurate in diagnosing true appendicitis for patients in the training data. This way, we can be as certain as possible that when the model outputs a negative diagnosis, it is not a false negative, but a true negative.

The mean and standard error of the target metrics were obtained by averaging the results from 50 outer-fold test sets (five-fold outer cross-validation repeated 10 times). The best model was chosen based on the mean specificity score while retaining maximum sensitivity.

Modeling was performed using Python (version 3.9.5, Python Software Foundation, Wilmington, DE, USA) and R Programming Languages (R Core Team, 2023, Vienna, Austria). Python packages used were “numpy”, “pandas”, “scikit-learn”, and “xgboost”. Within the R ecosystem, “dplyr”, “tidyr”, “purrr”, “ggplot2”, “plotly”, “tidymodels”, “ranger”, “xgboost”, and “fastshap” libraries were used. For random forest models, tuned hyperparameters were the number of variables to possibly be used for splitting at each node, and minimal node size to split at. For XGBoost, aside from the two hyperparameters already mentioned, the learning rate, gamma, number of trees, subsample ratio and maximum tree depth was tuned as well.

### Feature importance and model explainability

The significance of individual features was evaluated using shapley additive explanation (SHAP) values^19^. The values were obtained using the “fastshap” package with 10,000 simulations. By focusing on a specific feature within many feature coalitions, Shapley values measure the impact of each feature on shifting the prediction from the average model prediction to the final model output. Consequently, these values facilitate the explanation of predictions from ML models.

### Statistical analysis

The normality of data distributions was tested using the Kolmogorov–Smirnov test. For two group comparisons, the significance of differences was assessed using the *t*-test for normally distributed data or the Mann–Whitney test for deviations from normality. For comparisons involving more than two groups, ANOVA was used when data from all groups was normally distributed, and the Kruskal–Wallis test otherwise. The Chi-squared test was used for non-numerical features. A p-value threshold of 0.05 was used when considering statistical significance. The R programming language was used for all statistical analyses and visualizations.

### Ethical approval

The Ethics Committee of the University Hospital of Split approved the study protocol (approval no: 500-03/22-01/188; date of approval: November 28, 2022). Informed consent was not applicable for this study.

## Results

### Patient characteristics

In total, 551 patients were included in model training and evaluation, among which 252 patients had uncomplicated appendicitis, 252 had complicated appendicitis, and 47 patients had a negative PHD. Patient features and characteristics are described in Tables 2, 3.

**Table 2:** Patient characteristics for continuous features.

Feature	Negative PHD (n = 47)	Uncomplicated (n = 252)	Complicated (n = 252)	p value*
Age (years)	11.63 ± 3.75	11.73 ± 3.63	11.75 ± 3.92	0.98
Height (cm)	153.32 ± 21.58	153.49 ± 20.34	155.96 ± 21.39	0.42
Weight (kg)	48.76 ± 19.58	46.57 ± 18.25	47.17 ± 20.12	0.46
BMI (kg/m^2^)	18.76 ± 4.47	18.91 ± 3.8	19.06 ± 3.8	0.87
Body temperature (°C)	36.9 (1.2)	37 (0.9)	37.5 (1.4)	< 0.001
Symptoms duration(h)	28 (31)	24 (15)	30 (24)	< 0.001
CRP (mg/L)	10.3 (47.35)	11.55 (23.3)	46.45 (66.25)	< 0.001
Sodium concentration (mmol/L)	140 (2)	139 (3)	137 (4)	< 0.001
WBC count (10^9^/L)	11.5 ± 4.61	13.36 ± 4.36	16.58 ± 5.07	< 0.001
Lymphocytes (%)	15.6 (14.65)	13.8 (11.18)	7.95 (5.83)	< 0.001
Neutrophils (%)	76.9 (10.55)	79.3 (12.2)	84.9 (6.62)	< 0.001
Thrombocytes (10^9^/L)	276.15 ± 71.86	274.66 ± 67.76	289.93 ± 72.07	0.05
NLR	5.06 (6.22)	5.66 (5.3)	10.64 (8.66)	< 0.001
TLR	1.58 (0.64)	1.5 (0.96)	2.19 (1.42)	< 0.001
RDW (%)	12.7 (0.9)	13 (1)	12.8 (1)	0.07
MCHC (g/L)	343.5 (11.25)	343 (14)	345 (11.25)	0.15
MPV (fL)	8.5 (2.9)	8.1 (2.13)	8.3 (2.05)	0.86

Data presented as mean ± SD or median (IQR).

*BMI* body mass index, *CRP* C-reactive protein, *WBC* white blood cell, *NLR* neutrophil to lymphocyte ratio, *TLR* thrombocyte to lymphocyte ratio, *RDW* red blood cell distribution width, *MCHC* mean corpuscular hemoglobin concentration, *MPV* mean platelet volume.

*One-way ANOVA or Kruskal–Wallis test.

**Table 3:** Patient characteristics for categorical features.

Feature	Classification	Negative PHD (n = 47)	Uncomplicated (n = 252)	Complicated (n = 252)	p value*
Vomiting	No	30	142	75	< 0.001
	Yes	16	108	172	
Rebound tenderness	None	6	29	20	< 0.001
	Light	14	67	36	
	Medium	15	110	100	
	High	11	42	96	
Nausea	No	15	83	55	< 0.001
	Yes	30	161	199	
Sex	Female	20	167	168	0.005
	Male	27	85	84	
Pain migration	No	27	109	99	0.100
	Yes	19	140	140	

*Chi-squared test.

### Model for appendicitis prediction

The goal of this research was to develop a model that can reduce the number of negative appendectomies (appendectomies due to misdiagnosed appendicitis). Since surgical appendectomy is a low-risk procedure, it is considered the gold standard for treating acute appendicitis. Nevertheless, although rare, complications occurring during or after surgery are still possible.

As an alternative, conservative antibiotic treatment can be used. This approach results in low morbidity and mortality rates with a moderate recurrence rate. However, it's important to note that delaying surgical intervention can potentially lead to the development of complications. Since complications due to delayed surgery pose a more significant risk for the patient compared to surgical risks, surgery is usually the preferred option.

Taking the above facts into consideration, it is clear that the repercussion of false negative diagnoses is much higher than the cost of false positives. Consequently, while building a model for better identification of false positives, care should be taken to keep the number of false negatives as low as possible.

Following this logic, we chose to tune our model hyperparameters and thresholds with a custom metric that uses threshold shifting on the ROC curve to achieve the highest possible specificity score, while retaining maximum sensitivity. The threshold applied to the test set is determined as the second lowest threshold obtained from inner cross-validation. In terms of retaining high sensitivity on the test set, this is a more conservative approach compared to taking the mean threshold. In other words, we imposed a constraint that the model should be 100% accurate in diagnosing true appendicitis for patients in the training data. This way, we can be as certain as possible that when the model outputs a negative diagnosis, it is not a false negative, but a true negative.

#### Model characteristics

Labeling negative PHD findings as zeros, and positive (uncomplicated and complicated) as ones, we performed binary classification according to the above criteria using three different models—logistic regression, XGBoost boosted trees, and random forest (Table 4).

**Table 4:** Performance of three models in terms of predicting the presence of appendicitis.

Model	Sensitivity (mean ± SE)	Specificity (mean ± SE)
Random forest	0.997 ± 0.001	0.17 ± 0.01
XGBoost	0.9982 ± 0.0006	0.12 ± 0.01
Logistic regression	0.997 ± 0.001	0.052 ± 0.006

*SE* standard error.

**Figure 2:**
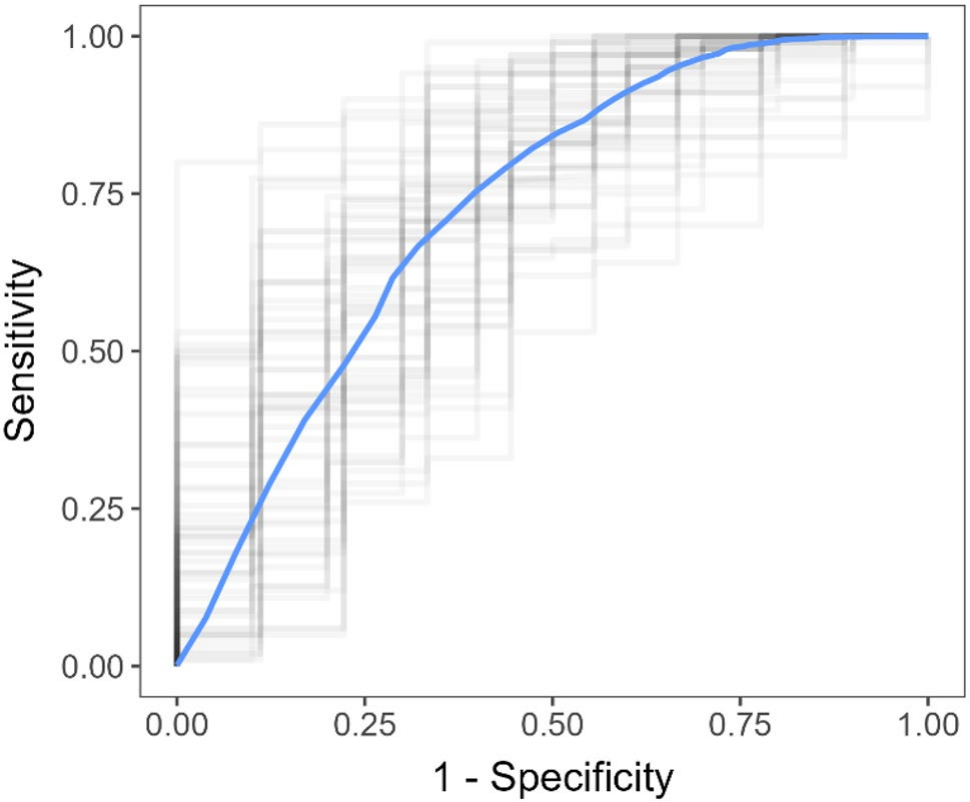
Random forest model average ROC curve (blue) and ROC curves of each outer fold (gray).

The best results were obtained with the random forest model, with a mean specificity and sensitivity of 0.17 ± 0.01 and 0.997 ± 0.001, respectively (Fig. 2). It is important to note that the false negative findings are practically all uncomplicated appendicitis cases, not complicated (gangrenous) ones. We can quantify this by calculating sensitivity separately for uncomplicated and complicated appendicitis patient groups (Table 5). The sensitivity was 0.995 ± 0.002 for uncomplicated appendicitis detection and 0.9996 ± 0.0004 for complicated appendicitis detection.

**Table 5:** Joint and PHD-specific sensitivities obtained from the random forest model.

PHD finding	Sensitivity (mean ± SE)
Complicated and uncomplicated	0.997 ± 0.001
Uncomplicated	0.995 ± 0.002
Complicated	0.9996 ± 0.0004

*SE* standard error.

**Figure 3:**
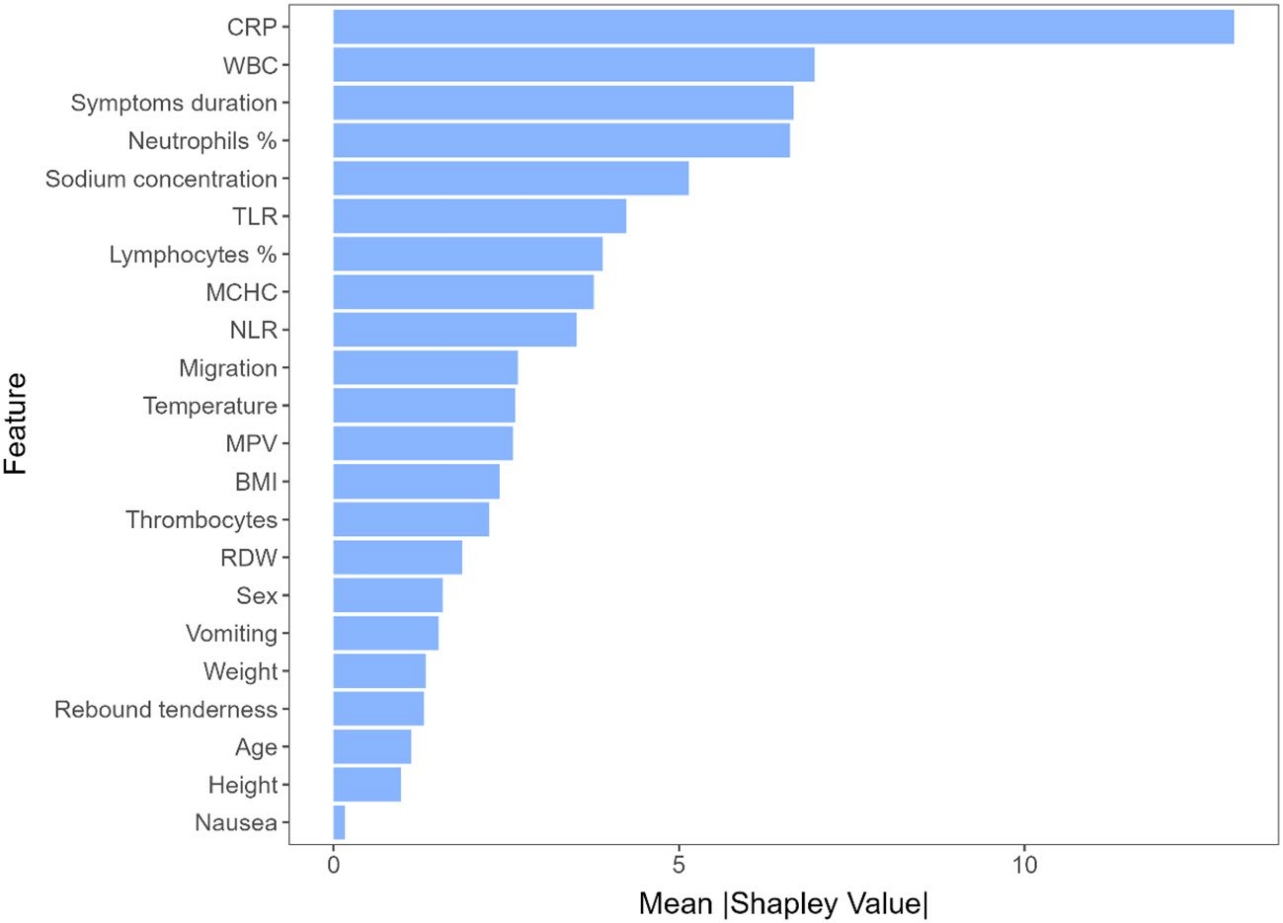
Feature importance based on approximate Shapley values for appendicitis prediction. *CRP* C-reactive protein, *WBC* white blood cell, *TLR* thrombocyte to lymphocyte ratio, *MCHC* mean corpuscular hemoglobin concentration, *NLR* neutrophil to lymphocyte ratio, *MPV* mean platelet volume, *BMI* body mass index, *RDW* red blood cell distribution width.

To better understand what the model has learned, we calculated Shapley values on one of the outer fold models to determine feature importance (Fig. 3). The top 10 most important features seem to have been CRP, WBC count, symptoms duration, neutrophils percentage, sodium concentration, TLR, lymphocytes percentage, MCHC, NLR, and pain migration.

#### Comparison to the AIR score

**Figure 4:**
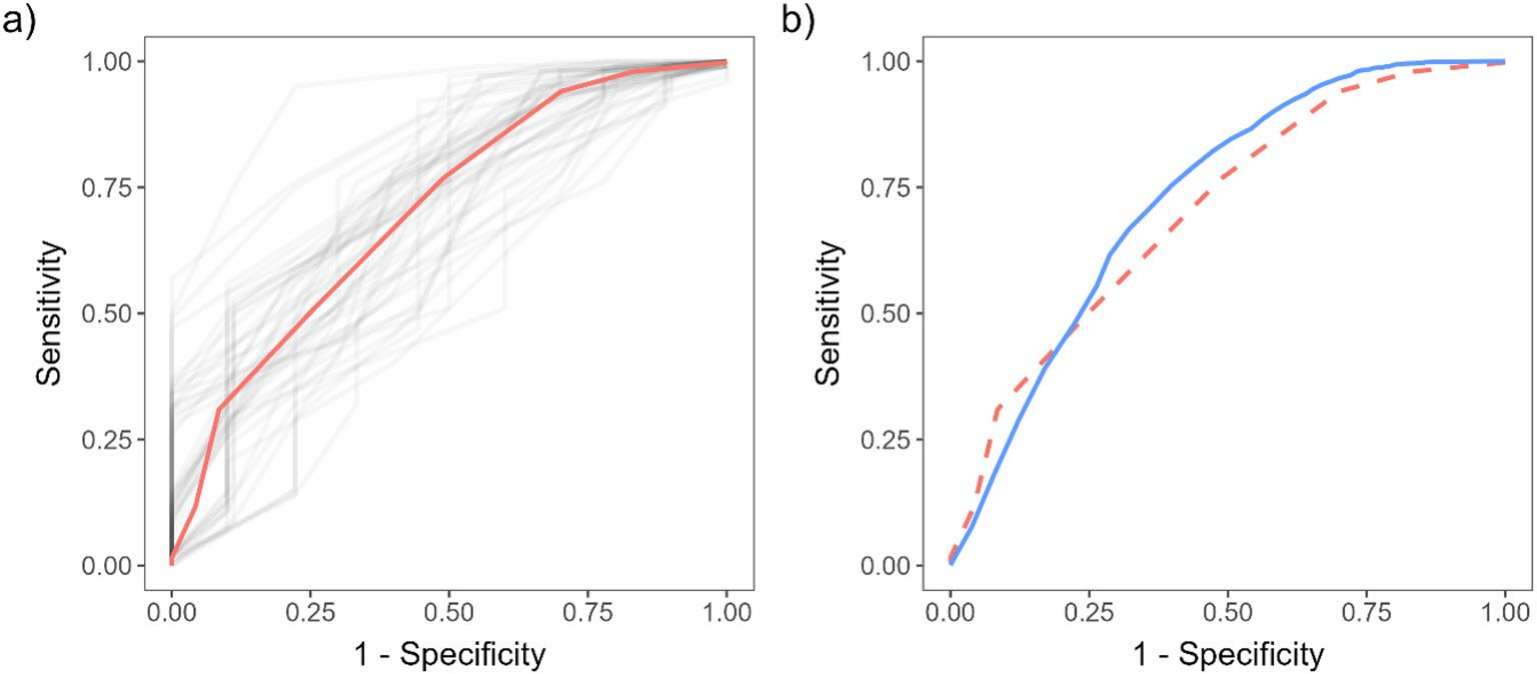
(**a**) AIR score average ROC curve (red) and ROC curves of each outer fold (gray). (**b**) Comparison of random forest model (full line, blue) and AIR score (dashed line, red) ROC curves.

To confirm the added value of our model, we also determined the sensitivities and specificities of AIR scores over all possible score thresholds by averaging values over the same 50 outer folds used in the evaluation of our model (Table 6, Fig. 4a). Performance for the threshold of AIR score greater than 0 in terms of sensitivity was similar to ours, but the specificity was 0. For an AIR score threshold set to values greater than 1, the specificity was similar to that of our model, but sensitivity was significantly lower (p = 5.674$\cdot$10^−14^, paired student’s *t*-test). Aside from the region of high sensitivity, our model outperformed the AIR score across most other sensitivity–specificity combinations as well (Fig. 4b).

**Table 6:** AIR score sensitivity and specificity over all possible score thresholds obtained by averaging values over the same five outer folds used in the evaluation of our model.

Threshold	Sensitivity (mean ± SE)	Specificity (mean ± SE)
0	0.9980 ± 0.0006	0 ± 0
1	0.979 ± 0.002	0.17 ± 0.01
2	0.940 ± 0.003	0.30 ± 0.02
3	0.768 ± 0.004	0.51 ± 0.02
4	0.507 ± 0.006	0.75 ± 0.02
5	0.310 ± 0.005	0.92 ± 0.02
6	0.117 ± 0.004	0.96 ± 0.01
7	0.016 ± 0.002	1 ± 0
8	0 ± 0	1 ± 0
9	0 ± 0	1 ± 0
10	0 ± 0	1 ± 0
11	0 ± 0	1 ± 0
12	0 ± 0	1 ± 0

*SE* standard error.

**Figure 5:**
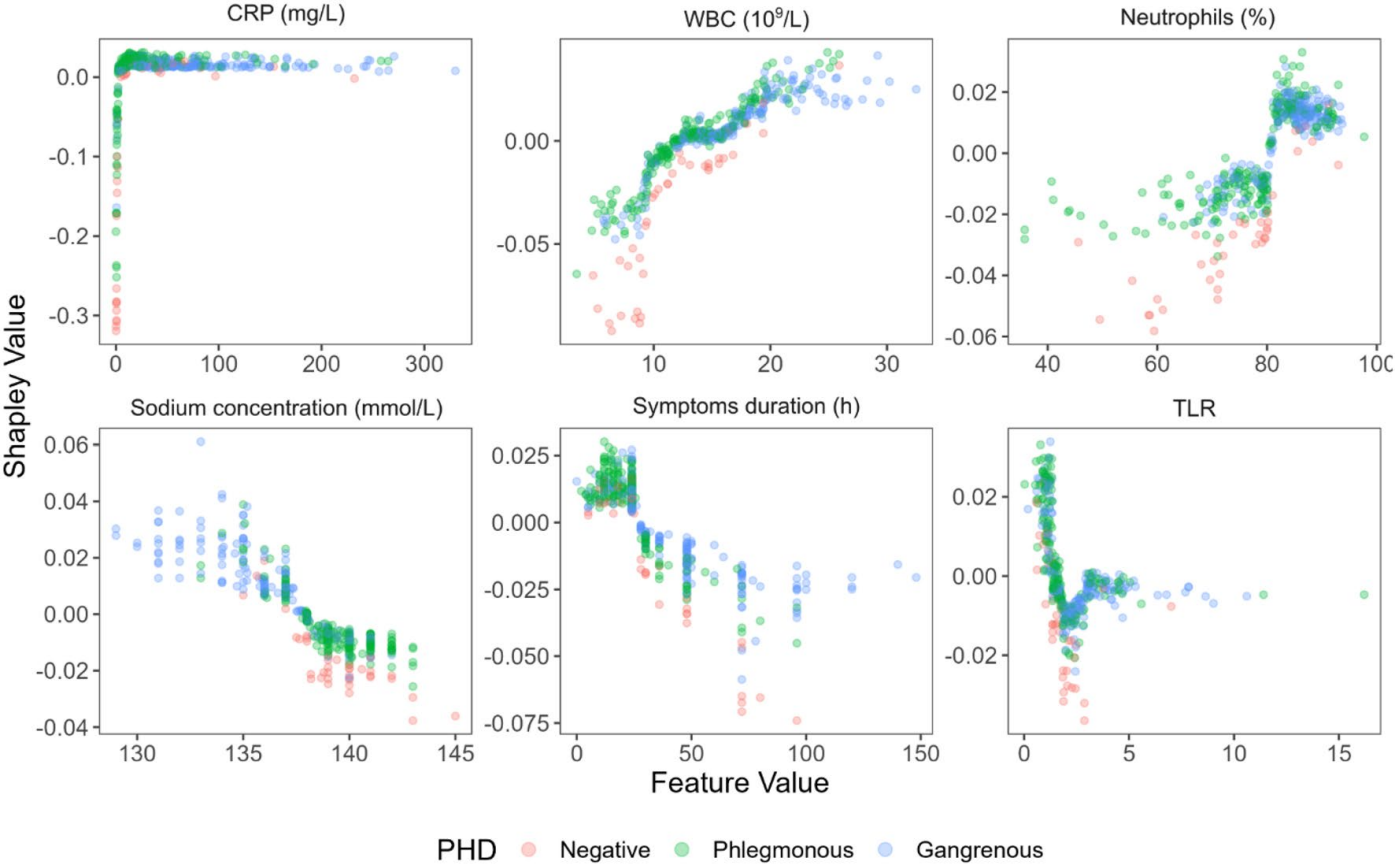
The relationship between key feature values and model predictions for the six most important features. *CRP* C-reactive protein, *WBC* white blood cell, *TLR* thrombocyte to lymphocyte ratio.

Figure 5 shows the relationship between the feature values and model predictions for the six most important features.

In terms of thresholds at which feature values increase the probability of appendicitis, we see a lot of similarity to AIR score cutoffs—around 10 mg/L for CRP, 10 × 10^9^/L for WBC count, and 80% for neutrophils percentage.

### Model for prediction of complicated appendicitis

**Figure 6:**
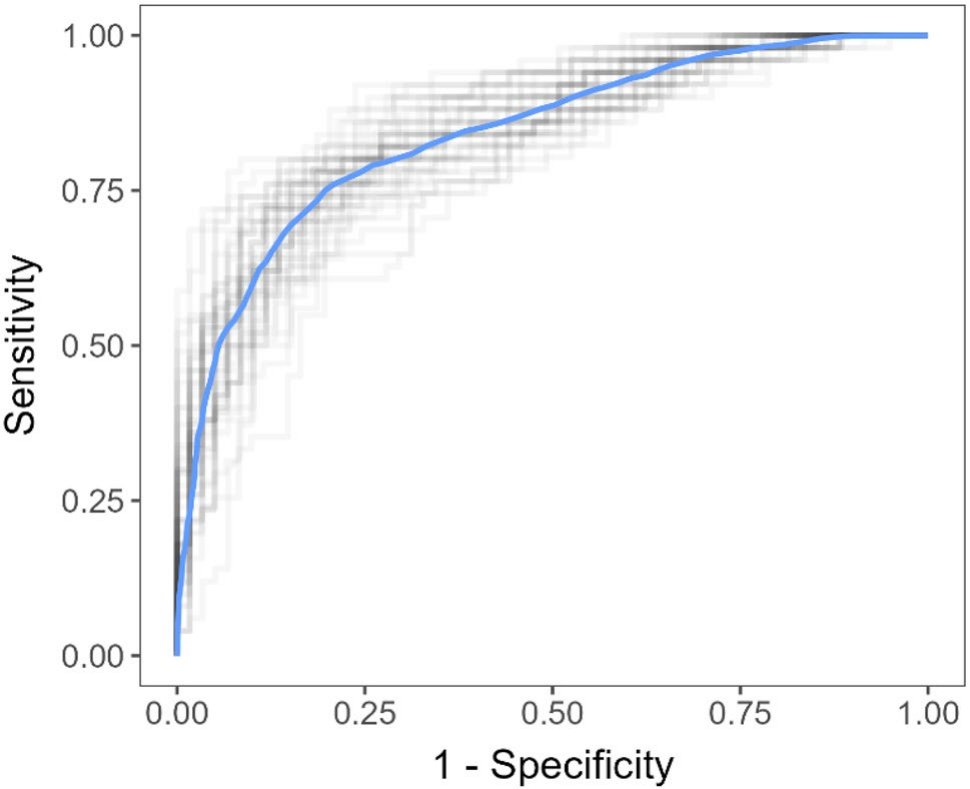
Random forest model average ROC curve (blue) and ROC curves of each outer fold (gray).

To account for the possibility of using conservative antibiotic treatment another classification model was used in which negative cases of appendicitis were bundled with uncomplicated ones and labeled as zeros, and only complicated ones were labeled as ones. Keeping everything else the same as in the above approach, the random forest model again provided the best predictions (Table 7), with a joint mean specificity of 0.129 ± 0.009 at the sensitivity of 0.994 ± 0.002 (Fig. 6). This model should be used when opting for the conservative approach to appendicitis treatment in which only patients with complicated cases of appendicitis undergo immediate surgery.

**Table 7:** Performance of three models in terms of predicting the presence of complicated appendicitis.

Model	Sensitivity (mean ± SE)	Specificity (mean ± SE)
Random forest	0.994 ± 0.002	0.129 ± 0.009
XGBoost	0.963 ± 0.008	0.26 ± 0.02
Logistic regression	0.9980 ± 0.0007	0.009 ± 0.004

*SE* standard error.

If we separate the specificities for negative and uncomplicated cases of appendicitis (Table 8), we obtain a specificity of 0.25 ± 0.02 for negative PHDs and 0.107 ± 0.008 for uncomplicated cases of appendicitis.

**Table 8:** Joint and PHD-specific specificities obtained from the random forest model.

PHD finding	Specificity (mean ± SE)
Negative and uncomplicated	0.129 ± 0.009
Negative	0.25 ± 0.02
Uncomplicated	0.107 ± 0.008

*SE* standard error.

**Figure 7:**
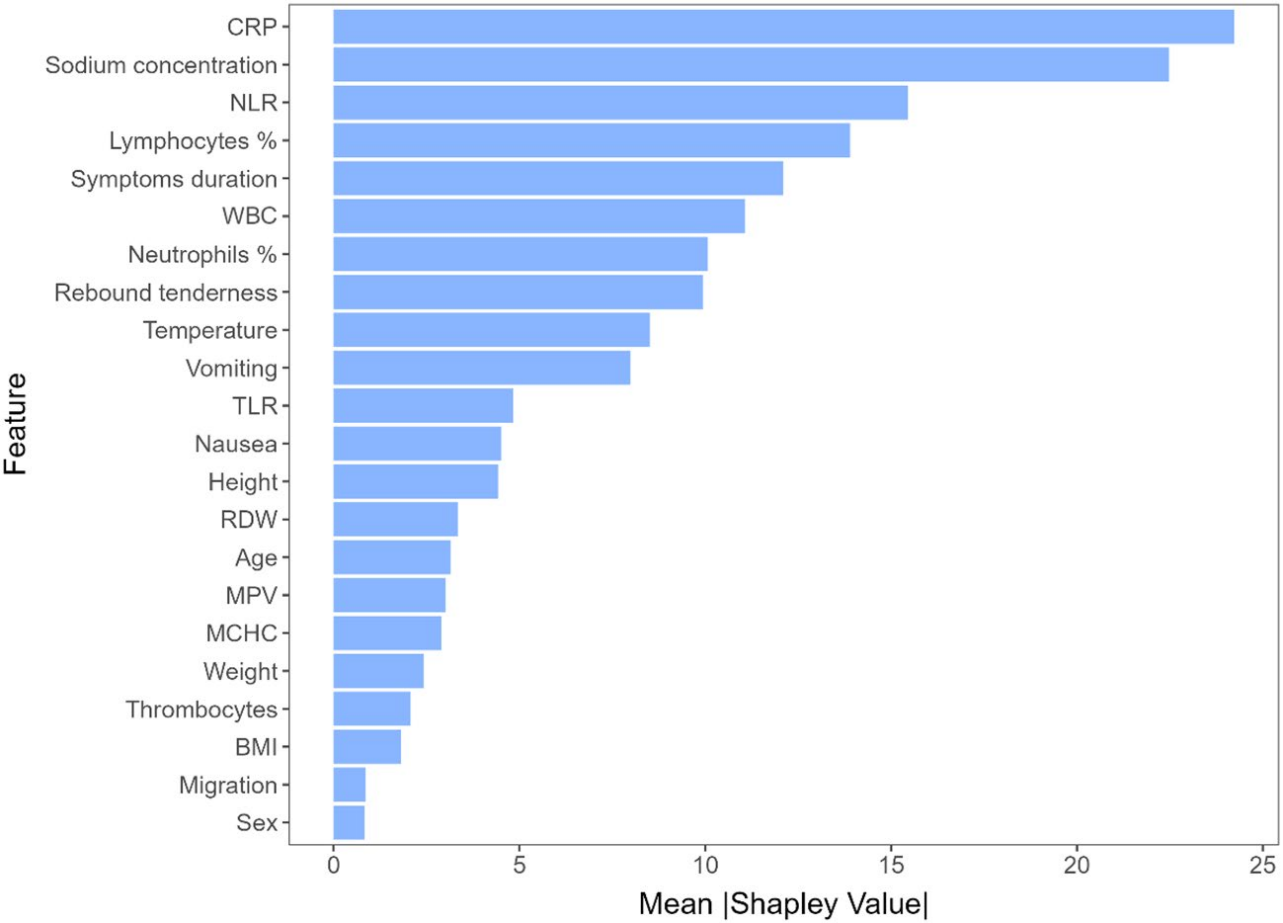
Feature importance based on approximate Shapley values for prediction of complicated appendicitis. *CRP* C-reactive protein, *NLR* neutrophil to lymphocyte ratio, *WBC* white blood cell, *TLR* thrombocyte to lymphocyte ratio, *RDW* red blood cell distribution width, *MPV* mean platelet volume, *MCHC* mean corpuscular hemoglobin concentration, *BMI* body mass index.

The top 10 most important features according to Shapley values seem to have been CRP, sodium concentration, NLR, lymphocyte percentage, symptoms duration, WBC count, neutrophils percentage, rebound tenderness, body temperature and vomiting (Fig. 7).

## Discussion

This study presents an ML model primarily aimed at the reducing negative appendectomies in the pediatric population. Although similar models have so far been developed to support the diagnosis of acute appendicitis, we believe that many of them have limited clinical usefulness as most models were steered towards higher specificity at the cost of sensitivity, inappropriately increasing the risk of misdiagnosing acute appendicitis. Additionally, some of the studies developed models with data from patients who did not have PHD findings, since they did not receive surgical treatment, which has significant impact on development of the ML models and results^10,20–23^. Furthermore, in some studies, the issue of finding the optimal balance between sensitivity and specificity was not discussed, and the authors believe that this is crucial if such models are to be used in clinical decision-making^21,23^.

The model was trained using data from pediatric patients who were already highly suspected of having appendicitis based on clinical judgment and who underwent surgery. In this high-risk group, all patients who actually had appendicitis were considered “true positives”, while those who turned out not to have appendicitis after surgery were “false positives”. There were no patients in the dataset who didn’t have surgery, so the model couldn’t learn to identify “true negatives” (no appendicitis, no surgery) or “false negatives” (appendicitis missed by the model). This model is intended for pediatric patients that a surgeon would typically recommend operating owing to high suspicion for appendicitis. In this population, our model would miss appendicitis in only 3 out of 1000 cases (99.7% sensitivity). In comparison, a hypothetical surgeon operating on all 1000 high-risk patients based on clinical judgment alone would correctly identify all appendicitis cases but would be wrong about the need for surgery 8.5% of the time (0% specificity). Our model achieved 17% specificity in this high-risk group, meaning that for every 1000 patients, it would prevent 17 unnecessary surgeries in children who didn’t actually have appendicitis, while still catching almost all true appendicitis cases (missing only 3). In summary, for pediatric patients already highly suspected of having appendicitis, our model could help prevent some unnecessary surgeries compared to current clinical judgment, while missing very few cases of true appendicitis.

It is important to note that there is a much higher chance that the false negative model predictions would be uncomplicated appendicitis cases, as evident from very high sensitivity in the case of gangrenous appendicitis detection (99.96%). This is relevant since some authors suggest that only complicated appendicitis should be immediately surgically treated, while uncomplicated cases can initially be treated conservatively using antibiotics (approximately 20–30% of these patients will end up in surgery)^24,25^. Therefore, provided that we apply antibiotic therapy in patients with high-risk but negative ML model results, the sensitivity could be increased even further.

This line of thought led us to test another classification model in which negative appendectomies were bundled with uncomplicated, and tested against complicated. Keeping everything else the same as in the above-noted approach, we obtained comparable sensitivity (99.4%), and somewhat lower specificity (12.9%). Theoretically, such an ML model could be used when opting for the alternative approach to appendicitis treatment in which only patients with complicated appendicitis undergo immediate surgery, whereas uncomplicated ones are initially treated with antibiotics. This approach would increase the number of false positives saved from surgery at the cost of assigning negative outputs to uncomplicated appendicitises. However, since the mislabeled uncomplicated appendicitises were those with the lowest probabilities of appendicitis according to the model, the proportion of patients who were labeled as zeros by the model and have to go to surgery after conservative treatment would most likely be lower than presumed 20%. In this regard, it is worth noting that increasing evidence suggests that not all patients with appendicitis will progress to perforation and that perforation occurs predominantly before the patients arrive at the hospital^8,26^. In addition, the mortality rate seems to be lower after perforation and larger after negative appendectomy than previously thought, indicating the importance of correct over rapid diagnosis^6^. Moreover, although we did not perform cost-benefit analysis, it is reasonable to suggest that reducing the number of unnecessary appendectomies would result in lower overall costs, even when accounting for missed cases.

In order to further establish the applicability of our appendicitis prediction model, we compared it to the AIR score^27^. To the best of our knowledge, this study is the first of its kind to make this comparison. For an AIR score threshold set to values greater than 1, the specificity was similar to that of our model, but at the cost of lower sensitivity (99.7% vs. 97.9%). On the other hand, if the cutoff for the low probability group was set at 5, as suggested by the authors of the AIR score, our model would significantly outperform the AIR score in terms of sensitivity, while still providing useful specificity values (99.7%/17% vs. 31%/92%)^27^. Nonetheless, as we observed a lot of similarity to AIR score cutoffs (around 10 mg/dL for CRP, 10 × 109/L for WBC count, and 80% for Neutrophils), the AIR score might be useful in settings where one needs a quick approximation of appendicitis or when only basic computational devices are available. However, the ability of ML models to shift the threshold values when necessary and capture nonlinear feature interactions results in superior model performance.

Several other authors developed ML models aimed at improving of diagnosis and management of patients with suspected acute appendicitis^10,22,28–31^. Unlike our study in which we aimed to preserve sensitivity as much as possible, other authors opted for high specificity and relatively low sensitivity despite similar AUC scores^32^. Furthermore, not all models were built based on definitive histopathological data for all patient cases, whereas some models were built on different populations (pediatric and non-pediatric population) or had significantly different numbers of false positive appendectomies, thus limiting our ability to compare the results^10,22^. Overall, it seems that different authors created models that are of similar accuracy to ours, but our model mostly stands out because of its aforementioned clinical relevance. On the other hand, studies defining the decision-making process in appendicitis using artificial neural networks (ANNs) yielded impressive results (91% sensitivity with 85% specificity, and 100% sensitivity with 97% specificity) but ANNs are burdened with significant data overfitting, and hence, without proper validation can lead to overly optimistic results^33–35^. For example, the study by Prabhudesai et al. involved training and testing an ANN on a limited dataset of only 60 patients^34^. The absence of methodologies such as bootstrapping and cross-validation in their approach significantly increases the risk of overfitting in the model^34,36^.

The present study is burdened by several limitations. Firstly, data was collected retrospectively. Secondly, the registry contains the data from only one hospital, and the results should thus be validated on other populations to provide better generalizability. Finally, the dataset was overall imbalanced towards positive appendicitis diagnosis, but this issue cannot inherently be resolved, given the restrictive nature of clinical decision-making for appendectomy referral. On the other hand, the greatest strengths of this study include the fact that the model is based on definitive histopathological reports, but does not require radiological techniques, such as ultrasound and CT, thus making the model widely available, even in underdeveloped regions. Finally, unlike most available ML models, our model maintained extremely high sensitivity, thus ascertaining that virtually every patient with acute appendicitis is adequately recognized as a candidate for appendectomy.

In conclusion, this study presents a machine learning (ML) model designed to reduce the number of unnecessary appendectomies in the pediatric population at high risk of acute appendicitis. The developed ML model aims to achieve high sensitivity to minimize the risk of missing an acute appendicitis diagnosis. Moreover, the model demonstrated superior diagnostic accuracy compared to the AIR score in this population. These findings suggest the potential use of ML models in assisting clinicians in making accurate decisions. Additionally, by presenting a model that predicts complicated (gangrenous) appendicitis cases, the study addresses the needs of centers that endorse performing emergency surgery only in patients with complicated appendicitis. In these centers, implementing such a model could be beneficial for appendicitis management.

Ultimately, the developed ML model could prevent 17% of high-risk patients from undergoing unnecessary appendectomies while maintaining a low rate of missed diagnoses (0.3%). However, to determine whether these models are beneficial in the diagnostic approach to patients with suspected appendicitis, they need to be externally validated in large cohorts.

## Data Availability

The data that support the findings of this study are available from the corresponding author, upon reasonable request.

## Abbreviations

AIR score: Appendicitis inflammatory response score
ANN: Artificial neural networks
AUC: Area under curve
BMI: Body mass index
MCHC: Mean corpuscular hemoglobin concentration
ML: Machine learning
MPV: Mean platelet volume
NLR: Neutrophil to lymphocyte ratio
pARC: Pediatric appendicitis risk calculator
PAS: Pediatric appendicitis score
PHD: Pathohistological diagnosis
RDW: Red blood cell distribution width
ROC: Receiver operator characteristic curve
SHAP: Shapley additive explanation
TLR: Thrombocyte to lymphocyte ratio
WBC: White blood cells
XGBoost: EXtreme gradient boosting

## References

[1] Abu Foul S, Egozi E, Assalia A, Assalia A, Kluger Y, Mahajna A (2019). Is early appendectomy in adults diagnosed with acute appendicitis mandatory? A prospective study. World J. Emerg. Surg..

[2] Becker C, Kharbanda A (2019). Acute appendicitis in pediatric patients: An evidence-based review. Pediatr. Emerg. Med. Pract..

[3] Ferris M, Quan S, Kaplan BS, Molodecky N, Ball CG, Chernoff GW (2017). The global incidence of appendicitis: A systematic review of population-based studies. Ann. Surg..

[4] Snyder MJ, Guthrie M, Cagle S (2018). Acute appendicitis: Efficient diagnosis and management. Am. Fam. Phys..

[5] Wang ZH, Ye J, Wang YS, Liu Y (2019). Diagnostic accuracy of pediatric atypical appendicitis: Three case reports. Medicine.

[6] Jumah S, Wester T (2022). Non-operative management of acute appendicitis in children. Pediatr. Surg. Int..

[7] Howell EC, Dubina ED, Lee SL (2018). Perforation risk in pediatric appendicitis: Assessment and management. Pediatr. Health Med. Ther..

[8] Di Saverio S, Podda M, Gerardi C, Cillara N, Fearnhead N, Gomes CA, Birindelli A, Mulliri A, Davies RJ (2020). Diagnosis and treatment of acute appendicitis: 2020 update of the WSES Jerusalem guidelines. World J. Emerg. Surg..

[9] Coccolini F, Fugazzola P, Sartelli M, Cicuttin E, Sibilla MG, Leandro G (2018). Conservative treatment of acute appendicitis. Acta Biomed.

[10] Marcinkevics R, Wolfertstetter PR, Wellmann S, Knorr C, Vogt JE (2021). Using machine learning to predict the diagnosis, management and severity of pediatric appendicitis. Front. Pediatr..

[11] Jukić M, Nizeteo P, Matas J, Pogorelić Z (2023). Trends and predictors of pediatric negative appendectomy rates: A single-centre retrospective study. Children.

[12] Pogorelić Z, Rak S, Mrklić I, Jurić I (2015). Prospective validation of Alvarado score and pediatric appendicitis score for the diagnosis of acute appendicitis in children. Pediatr. Emerg. Care.

[13] Pogorelić Z, Mihanović J, Ninčević S, Lukšić B, Elezović Baloević S, Polašek O (2021). Validity of appendicitis inflammatory response score in distinguishing perforated from non-perforated appendicitis in children. Children.

[14] Sim JZT, Fong QW, Huang W, Tan CH (2023). Machine learning in medicine: What clinicians should know. Singap. Med. J..

[15] Shin S, Austin PC, Ross HJ, Abdel-Qadir H, Freitas C, Tomlinson G (2021). Machine learning vs. conventional statistical models for predicting heart failure readmission and mortality. ESC Heart Fail..

[16] Chen C, Liaw A, Breiman L (2014). Using Random Forest to Learn Imbalanced Data.

[17] Velarde G, Sudhir A, Deshmane S, Deshmunkh A, Sharma K, Joshi V. Evaluating XGBoost for Balanced and Imbalanced Data: Application to Fraud Detection. arXiv preprint. https://arxiv.org/abs/2303.15218 [cs.LG]. Accessed 12 Dec 2023. (2023).

[18] More AS, Rana DP (2020). An experimental assessment of random forest classification performance improvisation with sampling and stage wise success rate calculation. Procedia Comput. Sci..

[19] Lundberg S, Lee SI. A Unified Approach to Interpreting Model Predictions. arXiv preprint. https://arxiv.org/abs/1705.07874 [cs.AI]. Accessed 12 Dec 2023. (2017).

[20] Phan-Mai TA, Thai TT, Mai TQ, Vu KA, Mai CC, Nguyen DA (2023). Validity of machine learning in detecting complicated appendicitis in a resource-limited setting: Findings from Vietnam. Biomed. Res. Int..

[21] Mijwil MM, Aggarwal K (2022). A diagnostic testing for people with appendicitis using machine learning techniques. Multimed. Tools Appl..

[22] Akbulut S, Yagin FH, Cicek IB, Koc C, Colak C, Yilmaz S (2023). Prediction of perforated and nonperforated acute appendicitis using machine learning-based explainable artificial intelligence. Diagnostics.

[23] Bhandarkar S, Tsutsumi A, Schneider EB, Ong CS, Paredes L, Brackett A, Ahuja V (2024). Emergent applications of machine learning for diagnosing and managing appendicitis: A state-of-the-art review. Surg. Infect..

[24] Yeh DD, Eid AI, Young KA, Wild J, Kaafarani HMA, Ray-Zack M, Kana’an T, Lawless R, Cralley AL, Crandall M, EAST Appendicitis Study Group (2021). Multicenter study of the treatment of appendicitis in America: Acute, perforated, and gangrenous (MUSTANG), an EAST multicenter study. Ann. Surg..

[25] Minneci PC, Mahida JB, Lodwick DL, Sulkowski JP, Nacion KM, Cooper JN (2020). Association of nonoperative management using antibiotic therapy vs laparoscopic appendectomy with treatment success and disability days in children with uncomplicated appendicitis. JAMA.

[26] Li J (2021). Revisiting delayed appendectomy in patients with acute appendicitis. World J. Clin. Cases.

[27] Andersson M, Andersson RE (2008). The appendicitis inflammatory response score: A tool for the diagnosis of acute appendicitis that outperforms the Alvarado score. World J. Surg..

[28] Reismann J, Romualdi A, Kiss N, Minderjahn MI, Kallarackal J, Schad M (2019). Diagnosis and classification of pediatric acute appendicitis by artificial intelligence methods: An investigator-independent approach. PLoS One.

[29] Akmese OF, Dogan G, Kor H, Erbay H, Demir E (2020). The use of machine learning approaches for the diagnosis of acute appendicitis. Emerg. Med. Int..

[30] Aydin E, Turkmen IU, Namli G, Ozturk C, Esen AB, Eray YN (2020). A novel and simple machine learning algorithm for preoperative diagnosis of acute appendicitis in children. Pediatr. Surg. Int..

[31] Stiel C, Elrod J, Klinke M, Herrmann J, Junge CM, Ghadban T (2020). The modified Heidelberg and the AI appendicitis score are superior to current scores in predicting appendicitis in children: A two-center cohort study. Front. Pediatr..

[32] Afzal B, Cirocchi R, Dawani A, Desiderio J, Di Cintio A, Di Nardo D (2023). Is it possible to predict the severity of acute appendicitis? Reliability of predictive models based on easily available blood variables. World J. Emerg. Surg..

[33] Hsieh CH, Lu RH, Lee NH, Chiu WT, Hsu MH, Li YCJ (2011). Novel solutions for an old disease: Diagnosis of acute appendicitis with random forest, support vector machines, and artificial neural networks. Surgery.

[34] Prabhudesai SG, Gould S, Rekhraj S, Tekkis PP, Glazer G, Ziprin P (2008). Artificial neural networks: Useful aid in diagnosing acute appendicitis. World J. Surg..

[35] Park SH, Han K (2018). Methodologic guide for evaluating clinical performance and effect of artificial intelligence technology for medical diagnosis and prediction. Radiology.

[36] Ghareeb WM, Emile SH, Elshobaky A (2022). Artificial intelligence compared to Alvarado scoring system alone or combined with ultrasound criteria in the diagnosis of acute appendicitis. J. Gastrointest. Surg..

